# Skin microbiota of first cousins affected by psoriasis and atopic dermatitis

**DOI:** 10.1186/s12948-016-0038-z

**Published:** 2016-01-25

**Authors:** Lorenzo Drago, Roberta De Grandi, Gianfranco Altomare, Paolo Pigatto, Oliviero Rossi, Marco Toscano

**Affiliations:** Clinical Chemistry and Microbiology Laboratory, IRCCS Galeazzi Orthopaedic Institute, Via R. Galeazzi 4, 20164 Milan, Italy; Medical Technical Sciences Laboratory, Department of Biomedical Science for Health, University of Milan, Via Mangiagalli 31, 20133 Milan, Italy; Clinical Dermatology, IRCCS Galeazzi Orthopaedic Institute, Via Galeazzi 4, 20164 Milan, Italy; Department of Biomedical Science for Health, University of Milan, Milan, Italy; SOD Immunoallergy Caraggi University-Hospital, Largo Giovanni Alessandro Brambilla, 3, 50134 Florence, Italy

**Keywords:** Psoriasis, Atopic dermatitis, Metagenomics, Skin microbiota

## Abstract

**Background:**

Psoriasis and atopic dermatitis (AD) are chronic inflammatory skin diseases, which negatively influence the quality of life. In the last years, several evidences highlighted the pivotal role of skin bacteria in worsening the symptomatology of AD and psoriasis. In the present study we evaluated the skin microbiota composition in accurately selected subjects affected by (AD) and psoriasis.

**Methods:**

Three first cousins were chosen for the study according to strict selection of criteria. One subject was affected by moderate AD, one had psoriasis and the last one was included as healthy control. Two lesional skin samples and two non-lesional skin samples (for AD and psoriatic subjects) from an area of 2 cm^2^ behind the left ear were withdrawn by mean of a curette. For the healthy control, two skin samples from an area of 2 cm^2^ behind the left ear were withdrawn by mean of a curette. DNA was extracted and sequencing was completed on the Ion Torrent PGM platform. Culturing of *Staphylococcus aureus* from skin samples was also performed.

**Results:**

The psoriatic subject showed a decrease in *Firmicutes* abundance and an increase in *Proteobacteria* abundance. Moreover, an increase in *Streptococcaceae*, *Rhodobacteraceae*, *Campylobacteraceae* and *Moraxellaceae* has been observed in psoriatic subject, if compared with AD individual and control. Finally, AD individual showed a larger abundance of *S. aureus* than psoriatic and healthy subjects. Moreover, the microbiota composition of non-lesional skin samples belonging to AD and psoriatic individuals was very similar to the bacterial composition of skin sample belonging to the healthy control.

**Conclusion:**

Significant differences between the skin microbiota of psoriatic individual and healthy and AD subjects were observed.

## Background

The largest organ of human body is the skin, which plays a pivotal role in protecting the host from pathogenic infections and penetration of harmful agents [1]. Before birth, the skin is completely sterile but after birth it is colonized by environmental microbes that are in homeostasis with the host [2, 3]. Moreover, after a vaginal delivery, fecal and vaginal microbes belonging to the mother’s bacterial microflora also colonize the skin of infants. The microbial community living on the human skin is called skin microbiota, and it is constituted by over 100 distinct species of bacteria [4]. Generally, we find four dominant phyla of bacteria colonizing the skin: *Actinobacteria*, *Proteobacteria*, *Firmicutes* and *Bacteroidetes* [5]. Several evidences exist about the pivotal role of bacteria in the development and persistence of atopic dermatitis (AD), a chronic itchy, inflammatory skin condition, very common in childhood. AD is considered to be a multifactorial disease, in which environmental and genetic factors contribute to its pathogenesis [1]. The incidence of AD has increased significantly in the last decades worldwide, leading the scientific community to hypothesize that the change in the lifestyle and nutrition may be involved in the development of aforementioned disease [5–7]. In the majority of individuals affected by AD, *Staphylococcus aureus* was found on the skin lesions. Furthermore, also the skin commensal *Staphylococcus epidermidis* has been observed to be increased with clinical disease activity, while bacteria belonging to the genera *Streptococcus*, *Propionibacterium* and *Corynebacterium* were increased only after a pharmacological therapy [8]. The skin microbiota dysbiosis may lead to a lack of immune system stimulation, together with an imbalance of type 1 T helper cells (T_H_1) and type 2 cells (T_H_2) activity, which might be involved in worsening of AD symptoms [9].

Psoriasis, instead, is a common inflammatory disease affecting 2–5 % of the population in industrialized countries. The disease is characterized by cutaneous inflammation and keratinocyte hyperproliferation, and it is often linked to severe complications, such as psoriatic arthritis [1]. Psoriasis has been hypothesized to result from a lack of immune tolerance to the skin microbiota in genetically predisposed individuals [10]. The aforementioned skin disease forms lesions on body often associated with beta-hemolytic streptococcal infection, where streptococcal superantigen leads T cell stimulation and expansion in the skin [11]. Several evidences highlighted the association between psoriasis and skin microbiota dysbiosis, underlying the relative abundance of *Corynebacterium*, *Propionibacterium*, *Staphylococcus* and *Streptococcus* in psoriasis plaques [12].

To date, the specific role of the skin microbiota in psoriasis and AD is still unknown, and it is unclear if the changes observed in the skin bacterial composition of individuals affected by psoriasis and AD are a cause or a consequence of alteration of the skin barrier, following the pathogenesis of aforementioned skin diseases. Certainly, the skin microbiota interacts with the host organism by producing several metabolites, which modulate cutaneous pro- and anti-inflammatory responses [13]. The current study used a next generation sequencing (NGS) approach to determine if some differences in the skin microbiota composition occur between individuals affected by psoriasis and AD, compared with a healthy control.

## Methods

### Study population

Three male first cousins aged 50 ± 3 years were chosen for the present study, according to specific and strict characteristics shared by all of them (Table 1). All subjects followed a diet rich in bifidogenic factors that promoted the growth of beneficial bacteria, such as bifidobacteria, and poor in allergenic foods that could worsening the symptomatology of psoriasis and atopic dermatitis. In particular, food containing vitamin A (peppers, carrots, spinach, basil, pumpkin), vitamin C (orange, lemon, and kiwi), folic acid (legumes, cereals, lettuce, asparagus), zinc (figs, sunflower seeds, potatoes) and omega-3 (fish) were allowed. In contrast, strawberries are often associated to allergies, in particular to atopic dermatitis, while tomato may lead to bowel dysfunction and it is not recommended for atopic subjects. Therefore, these foods were excluded from the diet of subjects enrolled in the study. Moreover, shellfish, cheese, fatty foods and red meat were not recommended (Table 1).

**Table 1:** Subjects’ selection criteria

Subjects’ characteristics	
Sex	Male
Age	50–53 years old
Subjects relationship	All individuals were first cousins
Diet	A Mediterranean diet was followed for 1 month before the day sampling. Pollen- and allergen- associated food, such as apple, hazelnut, celery, strawberries, shellfish and read meat were excluded from diet. Furthermore, milk intake was not recommended. Foods and beverages allowed were: bread, potatoes, vegetables, fresh fruit, meat and meat products, fish and fish products, eggs, edible fat, coffee, tea and soft drinks
Lifestyle	All individuals lived in the same neighborhood. No sport or daily exercises were practiced during the study period. No travel or excursion were carried out for at least 1 month before the day of sampling
Occupation	All subjects had a sedentary office work
Sexual activity	No sexual activity for 2 weeks before the day of sampling
Clothing	All individuals used only cotton clothes for all the study period
Pharmacological therapy	No antibiotic therapy was administered for at least 1 month before the day of sampling and they were not subjected to any kind of pharmacological therapy
Probiotic therapy	No probiotic therapy was administered for at least 1 month before the day of sampling
Personal care	All subjects used Cetaphil, a free-preservatives soap, once a day for 1 month before the day of sampling. Cetaphil is an oil-in-water petrolatum-based cream used to treat dry skin and often recommended for the management of AD. Moreover, no skin perfume or cream was used during the study
Others	No allergy to food, dust, pollen, grasses and drugs was present The subjects enrolled in the study shared no genetic diseases All subjects had not pets and/or contacts with any kind of animals All individuals were nonsmokers and not subjected to passive smoking The day of sampling took place the same day for all subjects enrolled in the study. The study was conducted during the winter season, in order to avoid excessive sweating

The selection criteria were chosen in agreement with dermatologists of the Clinical Dermatology Unit of IRCCS Galeazzi Orthopaedic Institute in Milan, Italy, where the study was conducted. One subject was affected by moderate AD, one had psoriasis and the last one was included as healthy control. The inclusion criteria for atopic dermatitis were moderate AD according to Hanifin and Rajka [14], with predominant rough and fissured skin as well as pruritus for at least 2 months. The inclusion criteria for psoriasis, instead, were the presence of psoriatic erythematous patches and the evaluation of psoriasis area and severity index (PASI) score, a tool used for the measurement of psoriasis severity. In particular, the PASI score of psoriatic subject enrolled in the study was 20.0, while the SCORAD for the AD subject was 32.46, underlying the presence of a moderate psoriasis and a moderate AD, respectively. Furthermore, subjects affected by psoriasis and AD had no concomitant diseases and both healthy and psoriatic individual had no any history of atopic dermatitis in childhood. The primary exclusion factors were the presence of chronic dermatosis such as seborrheic dermatitis, contact dermatitis, nummular eczema, ichthyosis, an immunodeficiency or any other immunological disorder, scabies, cutaneous fungal infection, HIV-associated skin disorders, malignant diseases, T-cell lymphoma, Letterer-Siwe disease, progressive systemic diseases, serious internal diseases (e.g., serious decompensated diseases of the heart, liver, and/or kidneys, or diabetes mellitus). The study was conducted according to ICH guidelines for Good Clinical Practice. All procedures followed were in accordance with the Declaration of Helsinki of 1975, as revised in 2000 and 2008. The study was approved by the Ethic Committee and Scientific Direction of the IRCCS Galeazzi Orthopaedic Institute. Subjects enrolled in the study provided verbal informed consent, which was carefully recorded in the study worksheets, to participate in the present study. Two lesional skin samples from a damaged area of 2 cm^2^ behind the left ear were withdrawn by mean of a curette from AD and psoriatic subjects. Moreover, two non-lesional skin samples were taken from the same area from AD and psoriatic individuals and used as internal control. For the healthy control, instead, two skin samples from an area of 2 cm^2^ behind the left ear were withdrawn by mean of a curette. Samples were placed in sterile Petri dishes (one dish for each subject’s sample) and stored at 4 °C until analysis. Of the two samples, one was used for metagenomics, and the other for cultural analysis. None of the individuals enrolled in the study was currently receiving specific therapy for psoriasis and AD (i.e. methotrexate, tacrolimus or pimecrolimus). No additional drugs (i.e. corticosteroids, anti-histamines) were used by subjects enrolled in the study. In particular, during the month before the day of sampling, the psoriatic individual was subjected to specific clinical investigations, such as screening for latent tuberculosis and rheumatoid arthritis, to determine the appropriate systemic therapy to be administered after the sampling. During this period, psoriatic individual could use lanolin, a skin protector, if necessary. The study was carried out after obtaining informed consent from all subjects, and in line with the guidelines for experimental studies on humans applicable within our Institute.

### DNA extraction

Total DNA was extracted from skin samples using the Genomic DNA Mini Kit (Tissue) following the manufacturer’s instructions (Geneaid, Italy). The protocol included an initial mechanical disruption step with a micropestle, followed by an enzymatic lysis incubation for 30 min at 60 °C.

## 16S rRNA gene amplification

Partial 16S rRNA gene sequences were amplified from extracted DNA using the 16S Metagenomics Kit (Life Technologies, Italy) that is designed for rapid analysis of polybacterial samples using Ion Torrent sequencing technology. The kit includes two primer sets that selectively amplify the corresponding hypervariable regions of the 16S region in bacteria: primer set V2–4–8 and primer set V3–6, 7–9. The PCR conditions used were 10 min at 95 °C, 30 cycles of 30 s at 95 °C, 30 s at 58 °C and 20 s at 72 °C, followed by 10 min at 72 °C. Amplification was carried out by using a SimpliAmp thermal cycler (Life Technologies, Italy). The integrity of the PCR amplicons was analyzed by electrophoresis on 2 % agarose gel.

### Ion torrent PGM sequencing of 16S rRNA gene-based amplicons

The PCR products derived from amplification of specific 16S rRNA gene hypervariable regions were purified by a purification step involving the Agencourt AMPure XP DNA purification beads (Beckman Coulter Genomics, Germany) in order to remove primer dimers. From the concentration and the average size of each amplicon, the amount of DNA fragments per microliter was calculated and libraries created by using the Ion Plus fragment Library kit (Life Technologies, Italy). Barcodes were also added to each sample, using the Ion Xpress Barcode Adapters 1–16 kit (Life Technologies, Italy). Emulsion PCR was carried out using the Ion OneTouch TM 400 Template Kit (Life Technologies, Italy). Sequencing of the amplicon libraries was carried out on a 318 chip using the Ion Torrent Personal Genome Machine (PGM) system and employing the Ion PGM Hi-Q kit (Life Technologies, Italy) according to the supplier’s instructions. After sequencing, the individual sequence reads were filtered by the PGM software to remove low quality and polyclonal sequences. Sequences matching the PGM 3′ adaptor were also automatically trimmed. 16 rRNA sequences were then analyzed by Ion Reporter Software, which comprises a suite of bioinformatics tools that streamline and simplify analysis of semiconductor-based sequencing data. The 16S rRNA workflow module in Ion Reporter Software was able to classify individual reads combining a Basic Local Alignment Search Tool (BLAST) alignment to the curated Greengenes database, which contains more than 400,000 records, with a BLAST alignment to the premium curated MicroSEQ ID database, a high-quality library of full-length 16S rRNA sequences. The final output of Ion Reporter Software was the identification of microorganisms and their abundance in the sample.

### Quantification of *Staphylococcus aureus* by means of cultivable method

Skin samples were transferred to 1.5 ml tubes and one ml of normal sterile solution (NaCl 9 g/l) was added. After homogenization to a homogeneous solution, samples were serially diluted in saline and appropriate dilutions were plated onto Mannitol Salt Agar (MSA), a selective medium for the growth of *Staphylococcus* spp, and incubated in aerobiosis for 48 h at 37 °C. All colonies of different morphology were identified according to: growth on selective medium, Gram staining, colony and cell morphology and the catalase and oxidase tests. The identification of *S. aureus* was performed by mean of RAPID Staph assay (Thermofisher, Italy). Finally, the percentage of occurrence of *S. aureus* in skin samples was calculated as follow:% *S. aureus* = [mean of CFU/cm^2^ (*S. aureus*)]/[mean of log10 CFU/cm^2^ (total staphylococci)] × 100.

### Statistics

Differences in *S. aureus* counts between the three individuals were evaluated by means of Student t test.

## Results

### Metagenomics analysis of skin microbiota in AD and psoriatic individuals and in healthy control

**Fig. 1:**
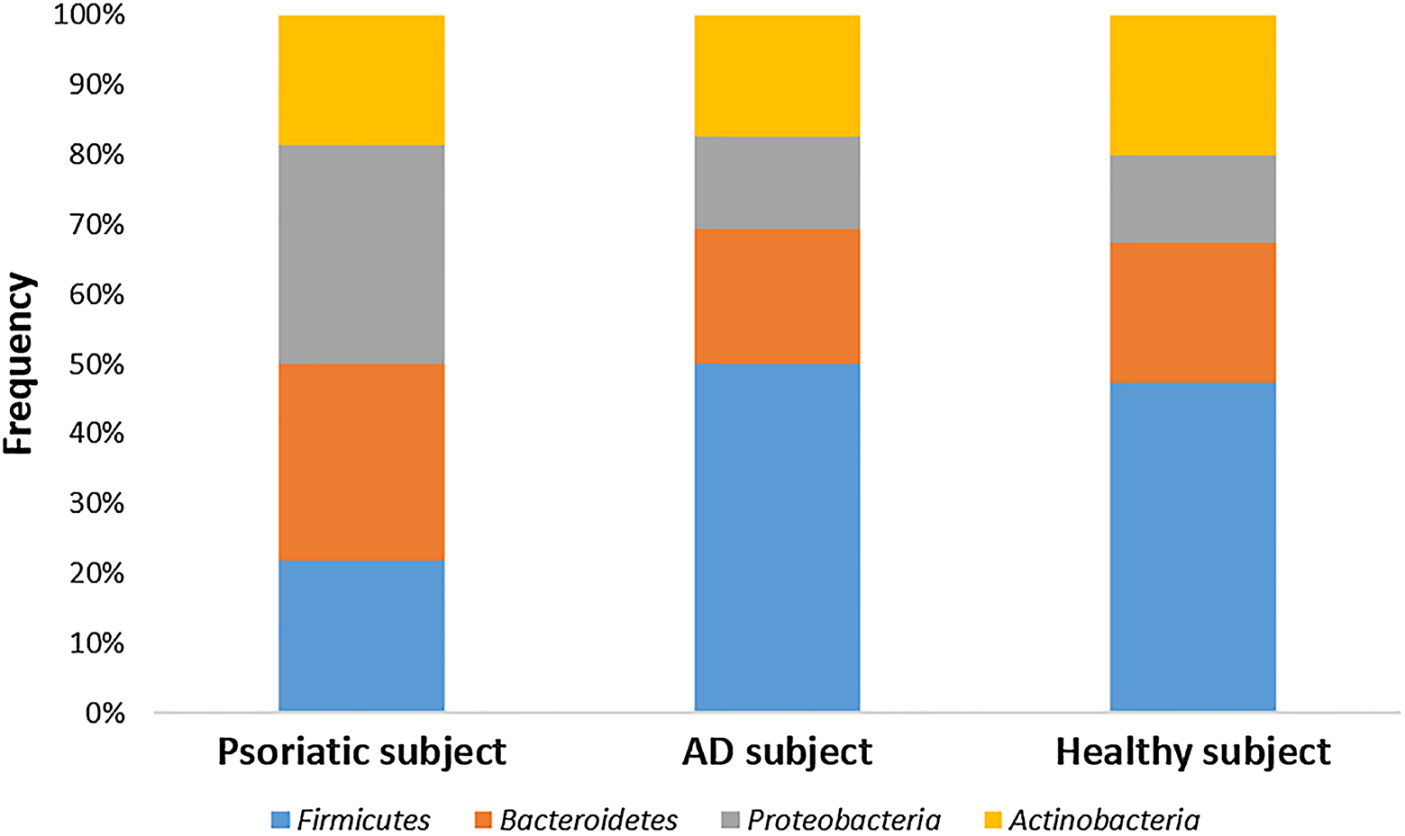
Relative abundances of bacterial phyla on lesional skin samples of psoriatic and AD individuals and in the skin of healthy control

**Fig. 2:**
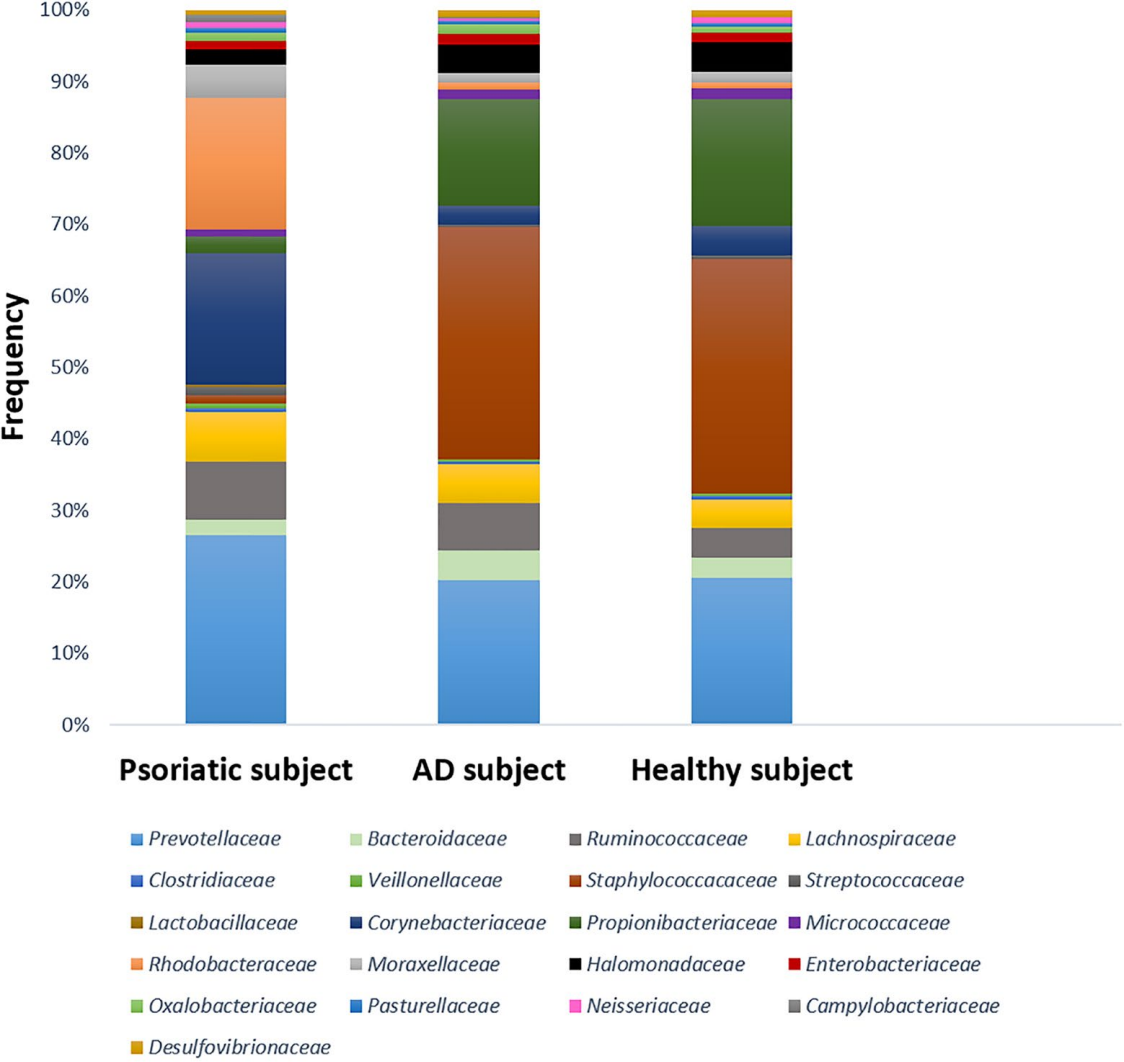
Relative abundances of bacterial families on lesional skin samples of psoriatic and AD individuals and in the skin of healthy control

**Fig. 3:**
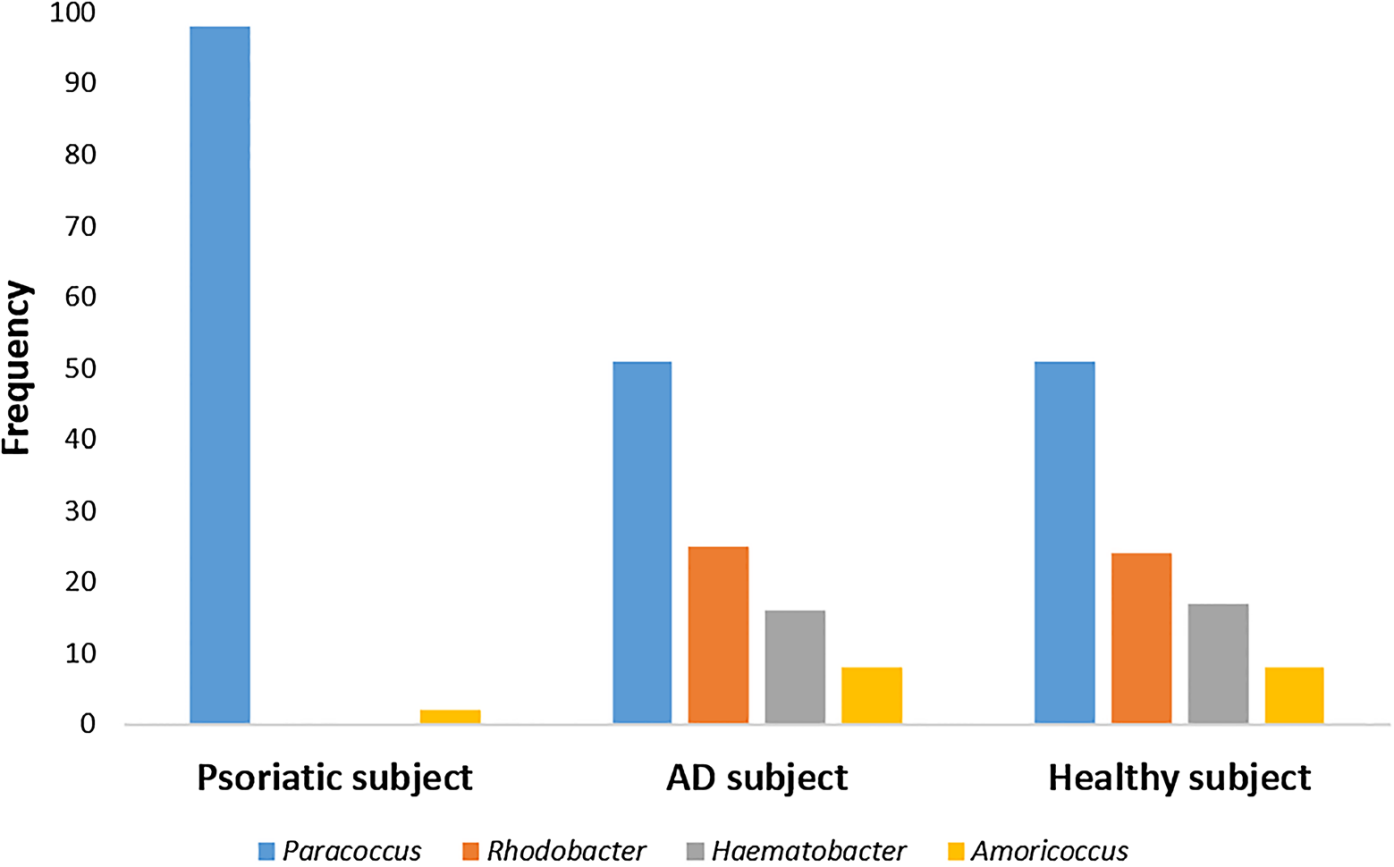
Relative abundances of bacterial genera among *Rhodobacteraceae* family on lesional skin samples of psoriatic and AD individuals and in the skin of healthy control

**Fig. 4:**
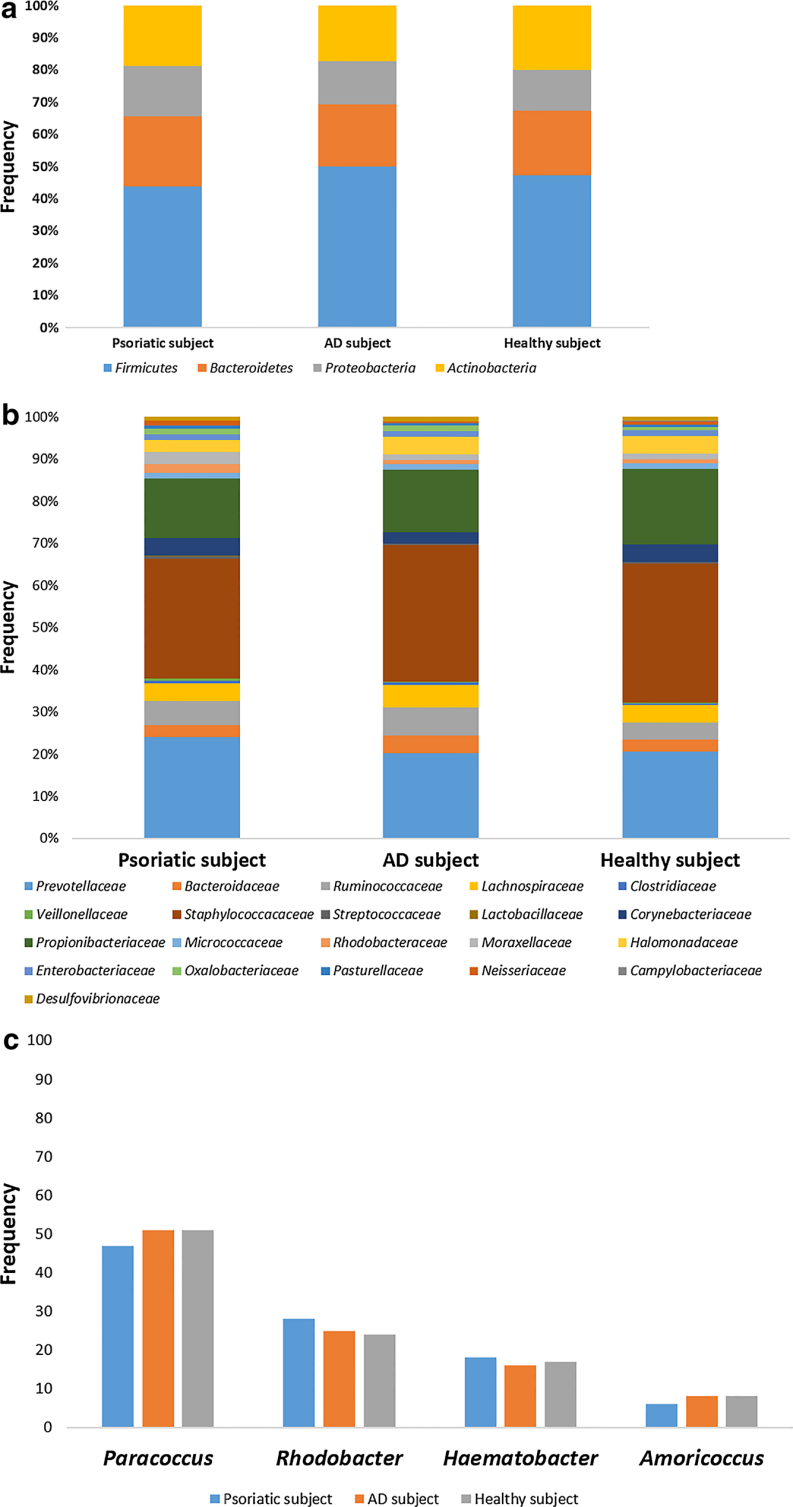
Comparison between non-lesional skin samples of AD, psoriatic and healthy subjects. Comparison between bacterial phyla composition (**a**); comparison between bacterial family composition (**b**); comparison between bacterial genera composition among *Rhodobacteraceae* family (**c**)

Figures 1, 2 and 3 represent the comparison between the cutaneous microbiota of damaged skin from AD and psoriatic individuals and the skin of healthy control. Comparing lesional skin samples from AD and psoriatic subjects to the skin sample of healthy control, the most prevalent phyla detected in all subjects included *Firmicutes*, *Bacteroidetes*, *Proteobacteria* and *Actinobacteria*. Subject affected by psoriasis showed a decrease in *Firmicutes* abundance and an increase in *Proteobacteria* abundance, if compared with AD patient and healthy individual (Fig. 1). A prevalence of *Proteobacteria* and *Bacteroidetes* over other phyla has been observed in psoriatic individual (Fig. 1). At family level, subject affected by psoriasis showed an increase in *Streptococcaceae*, *Rhodobacteraceae*, *Campylobacteraceae*, and *Moraxellaceae* if compared with control and AD patient (Fig. 2). However, even if psoriatic subject had a larger amount of *Rhodobacteriaceae*, a minor diversity in the family composition has been detected (Fig. 3). Indeed, in this subject the most abundant genus was *Parococcus* (i.e. >90 % of total *Rhodobacteraceae* total reads) while in healthy and atopic individuals also *Rhodobacter* and *Haematobacter* were found in skin microbiota (Fig. 3). Furthermore, psoriatic patient showed a decrease in *Staphylococcaceae* and *Propionibacteriaceae* abundance if compared with AD and healthy individual (Fig. 2). In particular, the total skin microbiota of psoriatic individual showed a decrease in *Propionibacterium acnes* abundance (i.e. <2 % of total reads), while in AD and healthy subject *P. acnes* was more abundant (i.e. >10 % of total reads). *Lactobacillaceae* were the population that contributed in the minor proportion to the overall skin microbiota (i.e. <0.5 % of total reads). Interestingly, no differences between the skin microbiota composition of AD patient and healthy control has been observed (Figs. 1, 2). Comparing non-lesional skin samples from AD and psoriatic subjects to the skin sample of healthy control, we did not observed any differences in the skin microbiota composition; indeed, bacterial phyla (Fig. 4a) and family (Fig. 4b) composition in AD and psoriatic individuals was very similar to that observed in healthy subject. Furthermore, in non-lesional skin samples of AD and psoriatic individuals we observed a similar composition at genus level among the *Rhodobacteraceae* family, if compared with the healthy control (Fig. 4c).

### Quantification of *S. aureus* in skin samples

**Fig. 5:**
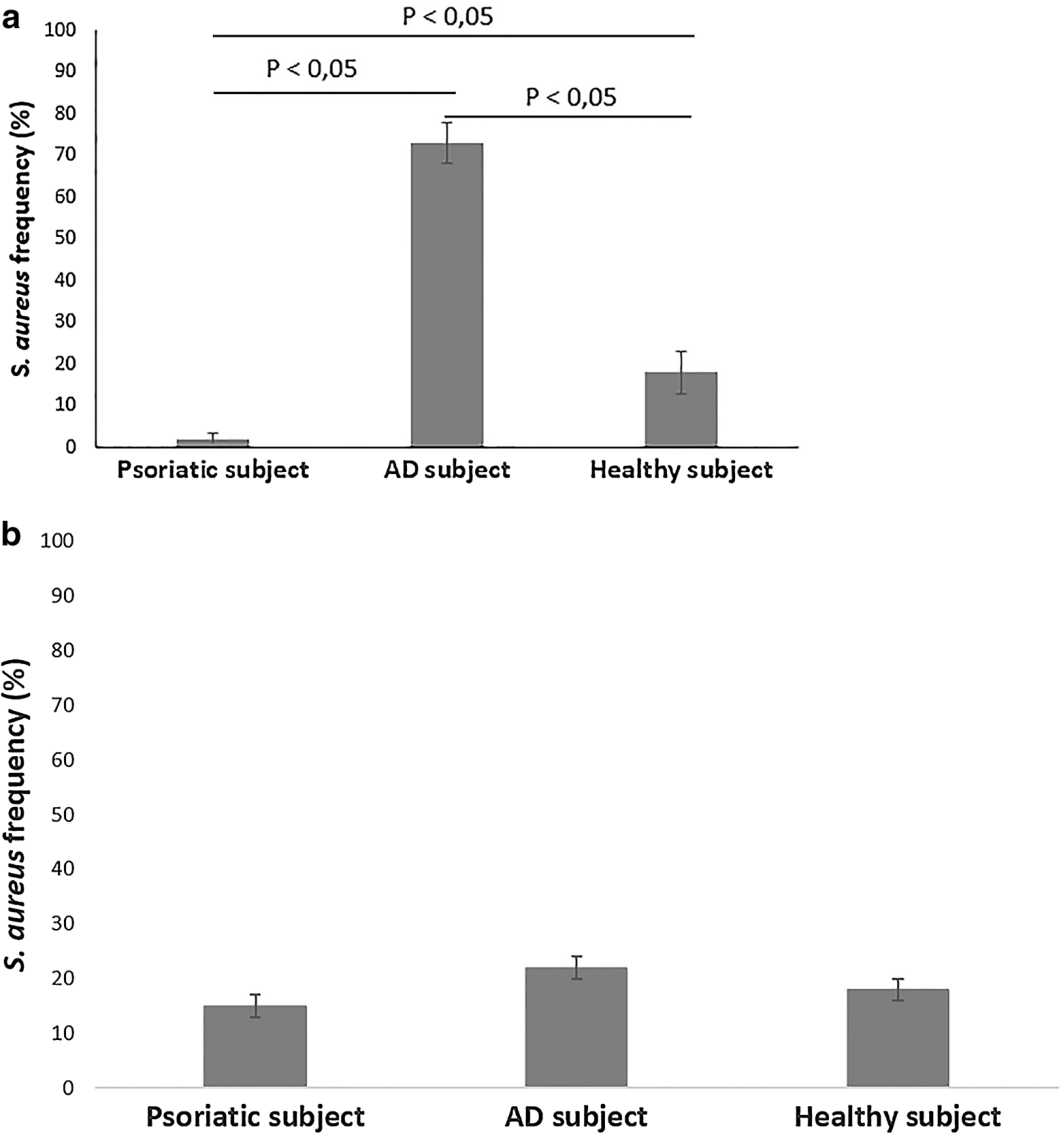
Frequency of *S. aureus* recovery on the lesional skin samples of psoriatic and AD individuals and on the skin of healthy control (**a**); frequency of *S. aureus* recovery on the non-lesional skin samples of psoriatic, AD and healthy individuals (**b**). Differences were considered statistically significant at p value <0.05

Patient affected by AD showed the larger abundance of *S. aureus* (Fig. 5a), with a frequency of 73 % of *S. aureus* on the total staphylococci load found on skin sample. By contrast, psoriatic individual had only a 2 % of *S. aureus* on his skin, while healthy control showed a frequency of 18 % (Fig. 5a). Moreover, no differences in *S. aureus* frequency were observed between non-lesional skin samples of AD and psoriatic subjects and healthy control (Fig. 5b).

## Discussion

This is the first study in which the skin microbiota of AD and psoriatic selected individuals subjected to the same lifestyle and environment factors has been compared. Indeed, we evaluated the skin microbiota of much selected subjects affected by psoriasis and AD, compared with the microbiota of a healthy related control. In particular, we analyzed samples from the area behind the left ear, evaluating the microbiota associated to this specific anatomical district. One of the main factor that seem to be involved in the development of allergic diseases is the mode of delivery; indeed, cesarean section has been observed to be associated with a moderately risk of allergic rhinitis, asthma and hospitalization for asthma [15]. One hypothesis concerns the composition of the gut microbiota, which is established early in childhood. Vaginal delivery leads to the first colonization of infant gut with maternal vaginal and fecal bacteria, while cesarean babies are deprived of this natural exposure and present a different gut microbiota [15]. The first steps of infant gut colonization play a pivotal role in normal tolerance induction, as well as in the development and homeostasis of the immune system. Therefore, cesarean delivery may lead to an increased susceptibility to atopic conditions. Interestingly, all subjects enrolled in the present study were born by vaginal delivery and no correlations with the development of AD and psoriasis can be suggested. However, significant differences have been detected in the abundance of several bacterial family in subjects enrolled in the study. *Propionibacteriaceae*, indeed, showed a different distribution among the three groups analyzed, as AD and healthy individuals had a larger abundance of aforementioned bacterial family and its major human species *Propionibacterium acnes*, if compared with the subject affected by psoriasis. *P. acnes* are dominant microorganisms in normal skin [16–19], and a decrease in the number of these microbes could be a reflection of disordered ecological niches that become inhospitable to these microorganisms, and probably play a pivotal role in the pathogenesis of psoriasis or could be involved in the disease worsening [17]. *P. acnes* could have a beneficial and protective role on the human organism, and in particular, at skin level, by mean of its immunomodulatory action in signaling human cells [18, 19]. It has been hypothesized that *P. acnes* could protect the human skin from the action of several pathogenic bacteria, and its decrease could lead to a reduction of the protective skin barrier [17]. However, to date it is not clear if the decrease observed in *P. acnes* abundance is a consequence of the inflammatory state of psoriasis, or if this reduction is involved in the pathogenesis of the aforementioned disease.

An increase in *Streptococcaceae* has been observed in psoriatic subject, confirming data of previous works, in which the presence of *Streptococcus* spp was linked to the pathogenesis of psoriasis [20, 21]. *Streptococcaceae* are highly relevant among the environmental factors that are involved in the development of psoriasis [21]. Different mechanisms, such as molecular mimicry, superantigens and the ability of streptococci for intracellular uptake and persistence in skin cells, may be involved in the pathogenesis of the disease. Furthermore, the skin microbiota associated to the subject with psoriasis showed a larger abundance of *Rhodobacteraceae*, in comparison to that of healthy and AD individual. Interestingly, a minor bacterial diversity has been observed in psoriatic sample, as *Paracoccus* was the predominant bacterial family detected. At the contrary, *Rhodobacter* and *Haematobacter* represented a significant part of the skin microbiota of AD and healthy subjects. *Rhodobacter* spp are able to produce a molecule, named lycogen, structurally similar to lycopene, which can acts as anti-inflammatory agent and as inhibitor of melanogenesis. This molecule is able to prevent the down-regulation of procollagen I and inhibit elevated production of NFκB, a transcription factor involved in cellular stress, which were elicited by UV-light exposure [22–24]. The absence of *Rhodobacteraceae* among the skin microbiota observed in psoriatic individual could be linked to the reduction of the physiological skin barrier integrity that is involved in the symptomatology and etiopathology of psoriasis.

Finally, our results did not underline a difference between the microbiota composition of AD individual and the healthy control, except for the higher frequency of occurrence of *S. aureus* in AD subject, even if a high abundance of *Staphylococcaceae* has been detected in both groups of individuals. Several evidences exist about the mainly role of *S. aureus* in the pathogenesis of AD [25–27]. In particular, the skin of AD subjects has been observed to be more frequently colonized by *S. aureus* if compared to that of healthy control. This microorganism is able to increase the skin inflammation by mean of specific toxins which act as superantigens and, as a consequence, they may induce monocytes and lymphocytes activation, increasing also the production of several pro-inflammatory cytokines [28, 29]. Interestingly, as the severity of AD lesions increased, also the load of *S. aureus* has been observed to become higher. Probably, the high abundance of *S. aureus* we detected in AD sample contributed to worsening the skin barrier damages, leading to the characteristic skin lesions that affected AD subject enrolled in the present study. Moreover, the pivotal role of *S. aureus* in AD was underlined by the low frequency of *S. aureus* in non-lesional skin sample of AD individual, compared to the lesional skin sample of the same subject. Interestingly, the microbiota composition of non-lesional skin belonging to AD and psoriatic individuals was very similar to the bacterial composition of sample from the healthy control. These findings suggested that the cutaneous dysbiosis we observed in psoriatic and AD subjects was directly linked to the skin damages that characterize psoriasis and AD. Moreover, bacteria belonging to *Parococcus* genera, which have been observed to be increased in psoriasis skin sample, seem to be involved in the formation of skin pustules and they may have a direct role in the maintenance and worsening of psoriatic skin lesions [30]. Cutaneous bacteria have already been observed to be directly involved in the pathogenesis of several skin diseases, in particular in psoriasis and AD, where patients show a significant difference between the microbiota composition of lesional skin and non-lesional skin [31]. *S. aureus*, for example, is thought to play a pivotal role in the pathogenesis of these diseases, being involved not only in the pathology onset, but also in its progression, leading to an exacerbation of inflammatory response [31]. Moreover, differences in bacterial composition often observed in different skin sites may be directly involved in the different severity of cutaneous lesions on specific skin areas.

Although, several contradictory data about the specific role of cutaneous microbiota exist due to different sampling techniques. Some studies analyze the microbiota composition using skin swabs, while others investigate the bacterial composition from the complete epidermis and dermis, leading to different results [31]. To date, there are no enough information about the specific role of the skin microbiota in cutaneous diseases, as it is not clear if the bacterial dysbiosis often associated to these dyseases is the leading cause or a consequence of the pathological status. Interestingly, several evidences underlined that also intestinal bacteria seem to be directly involved in the onset and in the maintenance of allergic diseases, suggesting the possibility of preventing or treating AD and psoriasis by influencing the intestinal microbiota. The gut dysbiosis, associated to disruption of intestinal barrier function, may lead to a significant increase in local and systemic inflammation that is often associated to allergic diseases [32, 33]. AD patients, in particular, showed several changes in the gut microbiota composition, as bifidobacteria were significantly lower in comparison to healthy subjects, while the number of staphylococci was significantly higher in AD individuals compared with controls. Consequently, a decrease of anti-inflammatory molecules due to the reduction of bifidobacteria and a parallel increase in pro-inflammatory molecules due to the proliferation of staphylococci in the intestinal environment, may lead to the worsening of inflammation status and symptomatology of allergic diseases [32].

## Conclusions

In conclusion, significant differences between the skin microbiota of psoriatic individual and healthy and AD subjects were observed. The majority of studies on AD- and psoriasis-associated skin microbiota focused their attention on the microbial population belonging to shoulder, finger, arm, forearm, elbow, abdomen, knee and leg, while we analyzed the area behind the left ear, highlighting how the skin area analyzed also could influence the microbiota composition in psoriasis and AD.

The forearm, for example, has been observed to be colonized mainly by microorganisms belonging to *Proteobacteria* and *Bacteroidetes* phyla, as well as the antecubital fossa that present also a high abundance of *Staphylococcaceae*. In popliteal fossa, instead, the most abundant microorganisms belong to the *Staphylococcaceae* family, while the inguinal crease is colonized mainly by *Corynebacterineae* [30]. Interestingly, the different skin sites present not only different kind of microorganisms, but they differ also for evenness and richness of bacterial communities [34]. Consequently, when a dysbiosis of the skin microbiota occurs, the kind of bacteria located on various skin sites can differently influence the onset and the progression of cutaneous diseases.

However, one limit of the present study was the low number of subjects enrolled, but our purpose was to minimize all variables that could influence the skin microbiota composition, applying stringent criteria of patient selection. Moreover, another vial of the present study was the lack of information about the family history of AD and psoriasis in the maternal or paternal line. However, the choice to enroll these three correlated-individuals with similar lifestyle have been taken to reduce the study variables, as previous studies enrolled subjects with different lifestyles and geolocation that could influence the skin microbiota composition. Further analysis will be needed to examine greater number of lesions in order to evaluate the connection of skin microbiota composition with the progression of inflammatory diseases. Furthermore, also patients with severe AD might be involved in the study to verify the correlation between the skin microbiota, and in particular the presence of *S. aureus*, and the disease severity.
